# Peripheral extremity surgery performed during the Syrian conflict – A scoping review

**DOI:** 10.1371/journal.pgph.0004116

**Published:** 2025-02-10

**Authors:** Anas Khan, Usman Ammar, Aula Abbara, Mahmoud Hariri, Ammar Darwish, Jude Alawa

**Affiliations:** 1 University Hospitals Sussex NHS Foundation Trust, Brighton and Hove, Brighton, United Kingdom; 2 School of Medical Sciences, University of Manchester, Manchester, England; 3 Faculty of Medicine, Imperial College London, St Mary’s Hospital, London, England; 4 Syrian Board of Medical Specialties, Gaziantep, Turkey; 5 David Nott Foundation, London, England; 6 Stanford University School of Medicine, Stanford, California, United States; Yale University School of Medicine, UNITED STATES OF AMERICA

## Abstract

The protracted Syrian conflict has devastated healthcare infrastructure across Syria with impacts on both the conflict and non-conflict related surgical needs of patients. This review summarises the literature related to peripheral limb surgery performed in Syria between March 2011 to January 2024 to determine the influence of the conflict on surgical capacity and provision. A systematic review based on Preferred Reporting Items for Systematic Reviews and Meta-Analysis extension for Scoping Reviews (PRISMA-ScR) guidelines was performed using six academic and one grey literature database. Studies that included clinical-epidemiological descriptions of procedures in orthopaedic, vascular and plastic surgery were included for quantitative and narrative analysis. The search resulted in 3951 papers, of which 7 met inclusion criteria. Studies varied widely in sample size (range: 25–3835; total: 7749), design, and outcomes. Procedures spanned orthopaedic (1285, 31%), vascular (741, 18%), and plastic surgery (2145, 51%), totalling 4171 cases. Common procedures included wound management (51%), external fixation (26%), and lower limb amputation (15%). There was prevalence of emergency over elective surgery, performed in rudimentary, secret field hospitals by practitioners lacking formal specialist training or aid-workers. Inadequate documentation and incomplete data capture, due to the conflict, hindered the availability of high-quality, comprehensive surgical datasets within Syria. The focus on emergency surgery is a natural consequence of the prioritisation required with conflict-related pathology, however poor resource availability resulted in challenges in providing basic surgical care. Further research is needed to address gaps in evidence regarding the current state of surgery, training needs, and the quality of care provided.

## Introduction

The Lancet Commission on Global Surgery highlights the unmet need that exists in low- and middle-income countries affected by prolonged conflict, particularly where health infrastructure has faced severe damage, and access to surgery is limited [1]. In Syria, the protracted conflict has forced more than half the pre-conflict population from their homes. More than half a million have been killed and tens of thousands are living with conflict related injuries [2–5]. The conflict has destroyed over 50% of medical facilities, severely compromising capacity for specialist surgeries [2]. This damage has affected different parts of Syria to varying degrees with areas outside of government control affected most [6].

The shortage of medical supplies during the Syrian conflict has reduced provision of both elective and emergency surgical services [7]. The current surgical capacity within Syria is not known, previous studies in similar settings have demonstrated that war cripples the provision of surgery and limits hospitals to providing basic procedures without anaesthetic support and oftentimes electricity and oxygen [8,9]. The reduced surgical capacity within Syria is demonstrated in several studies; a survey of Syrian refugees showed nearly 25% reported an untreated condition requiring surgery [4]. Similarly, an analysis of orthopaedic surgery in Istanbul, Türkiye of Syrian refugees found a significant increase in emergency procedures, which peaked in in 2016 (21% of all operations), perhaps linked to the Battle of Aleppo [10].

The advancement of weaponry, ballistics and explosives has meant that injury to the head or torso is usually lethal resulting in death in the field before any intervention is possible. The importance and rise of peripheral extremity surgery, including limb reconstruction and salvage (orthopaedics, vascular and plastic surgery) has been a large focus on modern 21^st^ century conflict for this reason [11–13]. Describing the state of limb surgery has become increasingly pertinent, for organisations and governments as well as individual surgeons for service planning and surgical training, particularly in the wake of the February 2023 earthquakes and more recent Israeli–Palestinian conflict which have resulted in further injuries [14,15]. Throughout the conflict, thousands of health workers have been forced to leave Syria leading to gaps in senior surgeons who are able to train junior colleagues [3,16]. In response to these challenges, surgeons and healthcare providers have been forced to learn new techniques to adapt to emerging circumstances, with the goal of maintaining quality of life and reducing disability from limb injury, [17].

This scoping review aims to summarise the academic and grey literature pertaining to peripheral limb and reconstructive surgery performed during the Syrian conflict. Specific objectives include developing an understanding of the types of procedures performed, equipment requirements, and the surgical challenges encountered. This knowledge could contribute to better preparedness for future conflicts and provide valuable insight into the surgical capabilities and nature of injuries occurring within the context of warfare, beyond the Syrian conflict.

## Methods

### Study Setting

All areas of Syria were included in this study though heterogeneity of the surgical needs in the different health systems is recognised. The conflict has resulted in fragmentation of health provision with different governance structures, population structures, needs and differing forms of funding and planning [7]. Northwest Syria, which includes the Idlib Governorate and parts of Aleppo Governorate, is under opposition control and shelters 4.2 million people, with over 67% being internally displaced people. Aleppo city experienced significant destruction during the Battle of Aleppo (2012–2016), including attacks on civilians, schools, and hospitals [18]. In Northeast Syria, Raqqa was heavily bombed and largely destroyed after being declared the capital of the Islamic State [19]. Presently, about two-thirds of Syria is under government control, where the health system largely resembles pre-conflict conditions but suffers from economic collapse, lack of investment and workforce shortages. Areas loyal to the government (e.g., Tartous, Lattakia, central Damascus) have experienced less conflict-related violence compared to other regions (e.g., Homs, Aleppo, Damascus suburbs).

### Search strategy and selection criteria

A systematic review of existing literature was conducted based on the Preferred Reporting Items for Systematic Reviews and Meta-Analysis extension for Scoping Reviews (PRISMA-ScR) guidelines [S1 PRISMA Checklist] [20]. A search was conducted using a combination of keywords such as “Syria”, “orthopaedic”, “plastic”, “vascular” and “surgery” and associated MeSH terms or synonyms where applicable. These searches were performed in the following databases: Cochrane library, MEDLINE, EMBASE, Scopus, Web of Science, Global health, and Google scholar. Additionally, the reference lists of relevant publications were manually reviewed to identify any key papers. The full search strategy is summarised in S1 File. Publications were required to have English full texts and be published from March 2011 as a surrogate for the start of the Syrian conflict until January 2024 when the search was performed. Papers that described clinical-epidemiological data of peripheral extremity surgery, with qualitative, quantitative, observational, or mixed methods were included. Procedures were required to be wholly performed in Syria. Abstracts, letters, correspondences and preprints were excluded. For article selection from grey literature sources, inclusion and exclusion criteria was similar without requirement for peer-review. The full inclusion and exclusion details is described in S1 Table.

### Study inclusion and data extraction

Initial search results were imported into web-based software, Covidence, for title and abstract screening based on the criteria above [21]. The software automatically removes exact duplicates. Two authors independently reviewed all studies, initially by title and abstract. Full texts of studies were subsequently retrieved and reviewed against the same criteria. Discrepancies were agreed by consensus. When the data from potentially eligible studies were insufficient, the corresponding authors were contacted and given 4 weeks to reply. Data were extracted by two authors into a spreadsheet including dates, location, participant demographics, study aims, procedures performed and conclusions. Values were presented as mean, unless otherwise specified.

### Assessment of the quality of included studies

Quality assessment was carried out by two authors independently using the Joanna Briggs Institute (JBI) critical appraisal tools specific to the study design [22].

## Results

**Fig 1:**
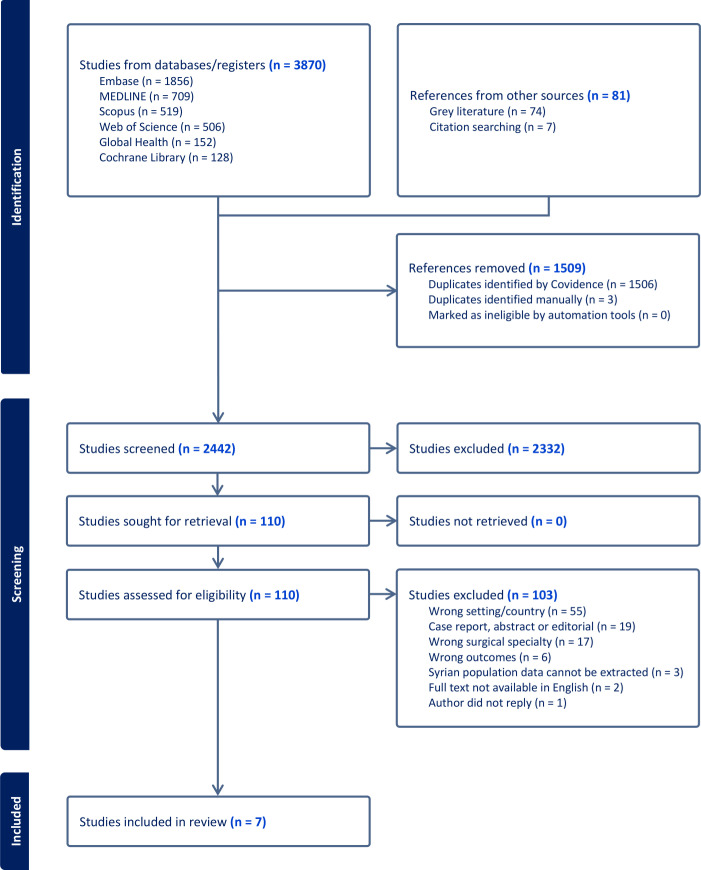
The Preferred Reporting Items for Systematic Reviews and Meta-analyses (PRISMA) flow diagram demonstrating sources of studies and reasons for study exclusion.

A total of 3951 papers were identified using the search strategy of which 110 underwent full text screening. Seven papers met the inclusion criteria with a total of 7749 participants (range 25–3835) who underwent 4171 peripheral limb surgical procedures

(Fig 1). The included studies where heterogenous with regards to study design, aims and outcomes making quantitative analysis challenging.

**Fig 2:**
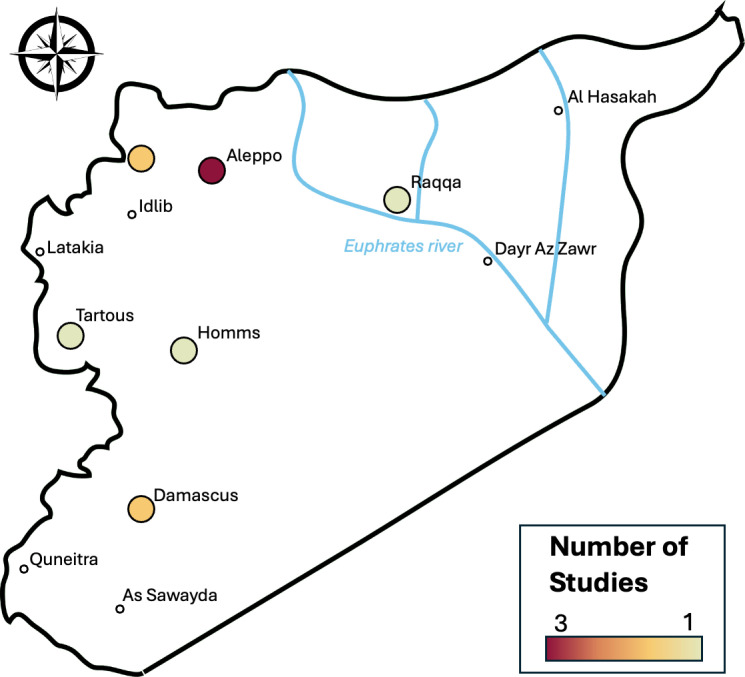
Map of Syria showing main cities and location that included studies were performed. Base layer map created using a file from Wikimedia Commons, the free media repository (source: https://commons.wikimedia.org/wiki/File:Syria_outline_map.png).

Pooled mean age was 43.8 years and 68% of total participants were male. Mean study duration was 2.5 years (range 8 days – 5 years). Two studies were performed in Aleppo [23,24], 2 in “North-West Syria” [25,26], 1 in Damascus [27], 1 in Raqqa [28] and 1 included data from several governorates across Syria [29] (Fig 2). Orthopaedic procedures were the most represented (5 studies) [23–25,28,29], followed by vascular [23,27,28] and plastics procedures [25,26,28] with 3 each.

The included studies’ main characteristics are summarised in Table 1. Four studies reported on procedures which were performed in “field hospitals” [23–26]. These included locations such as vaults of mosques, deserted palaces, caves and abandoned chicken farms [23,25]. These articles concluded with the need for effective communication, close proximity to the Turkish border and ensuring staffed were trained properly [23–26]. Okeeffe et al. aimed to describe the cases admitted to a MSF facility during and after an offensive period of conflict, whilst Aziz et al. described risk factors for diabetic foot infection and amputation [27,28].

**Table 1:** Table summarising all studies included in this systematic review.

Article, Year	Study date/duration	City	Aim	Sample, n	Age, *Mean ± SD Median (IQR)*	Male, %	Surgeries performed, n	Conclusion
Hasanin et al, 2013	NR “8 days”	Al-Bab (Aleppo)	Describe experience of field hospitals (mosque vault/deserted palace)	28	NR	NR	Vascular repair, 9 External fixation, 9	Establishing a field hospital necessitates: 1) effective communication 2) proximity to the Turkish border 3) training non-medical personnel for nursing roles 4) assembling a team of physicians specializing in various areas 5) implementing a triage plan
Trelles et al, 2015	Sept 2014–Jan 2014	Jabal al-Akrad, NW Syria	Describe quality, volume and type of surgery performed in field hospital (tent/abandoned chicken farm)	578	25 (21–32)	57	Wound care, 450 Orthopaedic (NS), 64	In conflict zones, meeting surgical needs involves: 1) Rapidly establishing surgical centres 2) Conducting procedures safely with skilled staff 3) Establishing referral networks 4) Ensuring reliable monitoring and evaluation
Alhammoud et al, 2019	Jul 2011 –Jul 2016	Aleppo (City)	Experience of field hospitals using external fixation for OSLBF	955	27.5 ± 11.0	92	External fixation, 967	External fixation is a dependable method for treating open long bone shaft fractures from high-energy trauma during conflict. It’s recommended for resource-limited areas.
Okeeffe et al, 2019	Jun 2017 –Marc 2018	Tal Abyad, Raqqa	Describe cohort of blast-wounded cases admitted to MSF DGH during offensive and post offensive periods	322	25 (17)	83	External fixation, 100 Amputation (NS), 65 Wound care, 1670 Orthopaedic (NS), 35 Vascular (NS), 23	Even after warring factions leave, there remains a significant risk of traumatic injury in conflict zones. As civilians return, they face a heightened danger from IEDs and ERWs, leading to a surge in blast injuries.
Aziz et al, 2020	Jan 2012 –Dec 2017	Damascus	Describe diabetic foot care and risk factors for amputation	2006	60	57	Major and minor lower limb amputations, 106 and 538	Diabetic foot patients are at higher risk of neglect during the conflict and present to podiatric and vascular surgeons with more advanced disease
Galhom et al, 2022	Oct 2012 –Mar 2013	NW Syria	Evaluate movable field hospitals for peripheral nerve injuries	25	26 ± 7.4	96	Nerve repair, 21 Nerve grafts, 4 External fixation, 6	In a war zone, a mobile field hospital offers surgical care for patients with peripheral nerve injuries from low-velocity shrapnel. Follow up for functional and electrophysiological assessment was not possible
Przepiórka et al, 2022	Jan 2019 –Dec 2019	Aleppo, Tartous, Damascus, Homms	Describe healthcare data in 2019 by ACN	3835	NR	NR	Orthopaedic (NS), 66	Patients lacked the most basic medical and surgical healthcare. Poor and unreliable data requires cooperation of healthcare provides, charities, humanists, and social workers.

NR = not reported, NW = North-West, NS = not specified, OSLBF = open shaft long bone fracture, MSF = Médecins Sans Frontières, DGH = district general hospital, IED = improvised explosive devices, ERWs = explosive remnants of war, ACN = Aid to The Church in Need.

Included articles present data from the earlier years of conflict and there were no publications from the last 5 years. Regarding the quality assessment, all studies were deemed to be suitable for inclusion. Poor response rates and lack of methods to address the poor follow up rates was the biggest areas of concern generally 9 (S1 Fig).

The 4171 reported procedures could be split into the following subspecialties: orthopaedic (n = 1285, 31%), plastic surgery (n = 2145, 51%), vascular surgery (n = 741, 18%) (Table 2). The most commonly reported procedures were wound care, including debridement, dressing or wound closure (n = 2120), external fixation for open fracture (n = 1082) and minor lower limb amputation (below the ankle) (n = 538). Alhammoud et al. in their report of management of open shaft long bone fractures (OSLBF) during the Syrian conflict found that the overall bone union rate, among 955 included patients, was 68% for patients receiving external fixation, with union after an average 5 months. There was no significant difference in rate between various types of external fixator device [24].

**Table 2:** Number of surgical procedures performed in Syria since the start of the Syrian civil war by surgical specialty.

Type of procedure	Procedures performed, n (%)
Trauma and Orthopaedic	1285 (30.8)
* External fixation*	*1082 (25.9)*
* Open reduction and internal fixation*	*22 (0.5)*
* Fixation with rod implant*	*16 (0.4)*
* Orthopaedic procedure (unspecified)*	*165 (4.0)*
Vascular surgery	741 (17.8)
* Vascular repair*	*9 (0.2)*
* Major amputation*	*106 (2.5)*
* Minor amputation*	*538 (12.9)*
* Amputation (unspecified)*	*65 (1.6)*
* Vascular procedure (unspecified)*	*23 (0.6)*
Plastic surgery	2145 (51.4)
* Wound care*	*2120 (50.8)*
* Primary end-to-end nerve repair*	*16 (0.4)*
* Sural nerve graft*	*4 (0.1)*
* Neuroma repair*	*5 (0.1)*
Total	4171 (100.0)

Reported perioperative (i.e., before, during or after surgery) complications includes deaths and wound infections. Two papers provided outcomes for intraoperative deaths [23,25]. Hasanin et al. and Trelles et al. reported 2 and 4 deaths for their respective field hospitals among 28 and 578 surgeries performed respectively. Overall, this equated to 1% mortality however neither a paper gave a breakdown by surgical speciality so intraoperative deaths related to orthoplastics alone is not known.

Regarding post-operative infections, these were mentioned in 3 papers. This included studies looking at external fixation for OSLBF (total = 404), a Médecins Sans Frontières (MSF) supported hospital in Raqqa (total = 322) and, and management of peripheral nerve injuries (total = 25), all in field hospitals. Infection rate was 16.7%, 10.6% and 12.0% respectively, providing a pooled mean post-operation infection rate of 15.0% [24,26,28]. Surgical specialty specific data were not provided by Okeefe et al. so their value represents post operative infections from abdominal and thoracic surgery as well. Of note, Okeeffe et al. also reported that the infection rate was not significantly different between the offensive and post-offensive period in Raqqa (8.3% vs 11.6%, p = 0.38) [28]. Galhome et al. found that deeper infections (8%) were more common than superficial infection (4%) following nerve injury repair [26].

One study reported on the costs of conflict related surgeries [29]. Przepiórka et al. reported on 3835 patients that benefited from *Aid to The Church in Need* (ACN) support in 4 cities in Syria in 2019. 78% of financial support went towards treatment of which 33% of this was surgical in nature. The team found that the cost of trauma was higher than non-trauma admission in the whole of Syria in 2019 ($351 ± 375 vs $60 ± 131, P < 0.05). Unfortunately, no breakdown is provided for the surgical procedures, which likely included other types of surgery (i.e., facial, abdominal, cardiac, etc) [29]. No other studies mentioned costs.

## Discussion

This is the first review of peripheral limb surgery performed during the Syrian conflict; despite large number of surgeries conducted during this time, there is limited number reported in academic literature. Studies are heterogenous, mostly reporting for a single site and often not disaggregated by type of surgery with limited reporting on complications, outcome, or costs. This review highlights the importance of implementing health information systems based on ICD–10 classifications which capture sufficient data that can support quality of care, support an understanding of the surgical needs in settings of conflict, how these change at different stages of conflict and where the surgical training gaps exist [30].

Included studies span diverse locations reflecting the widespread impact of the conflict on healthcare capacity and surgical provision. The studies from Aleppo mainly provide data from field hospitals in the initial years of conflict, coinciding with the Battle of Aleppo or the “mother of battles” [18,23,24]. Similarly, Okeefe et al. report MSF data from the offensive and post-offensive period in Raqqa, which culminated in the liberation of the city [28]. In summary, the data reported appears to be from intense periods of conflict during key events in this prolonged conflict, possibly explaining the predominance of emergency over elective surgery. The lack of data from the later years of the conflicts suggests that some de-escalation of military operations has occurred or that healthcare teams have shifted their focus away from research output.

A systematic review of 38 papers conducted in 2020 described traumatic injuries during the conflict; they found that there was a distinct pattern of injuries, mainly composed of gunshot wounds (GSW) rather than blast wounds when compared to other regional conflicts [31–33]. This review demonstrated the primary cause of amputation in Syria was diabetic foot disease (DFI), compared blast injuries in Iran and Afghanistan [34]. This discrepancy may be attributed to unique trauma profile of the Syrian conflict or limited access to diabetic care resulting in poor glycaemic control. These differences emphasise the need for detailed epidemiological studies to better inform wartime surgical and medical interventions.

Conflict-related injuries often necessitate urgent and prompt surgical management to prevent loss of life and support limb salvage. During times of conflict, the focus on emergency surgery is a natural consequence, with orthopaedic trauma management prioritised over routine care due to the severity of conflict-related injuries [31]. Elective procedures were rare, highlighting the destruction of healthcare infrastructure and a shortage of medical personnel. This scenario is further evidenced with surgeries that took place in makeshift, clandestine field hospitals, especially early in conflict when frontlines frequently shifted and secure areas were required to be set-up to deal with immediate injuries. These limited facilities demonstrated the adaptability and resilience required to provide surgical care in such challenging conditions [23–26]. Additional challenges emerged, such as the need for periodic evacuation of surgeons due to security threats, sometimes lasting up to 2 weeks [25]. Tragically, it was not uncommon for medical practitioners to become casualties, leading to situations where non-medical personnel had to undertake nursing duties [23,26].

Wound care emerged as the most frequently performed procedure. A critical initial step in managing injuries, wound care is paramount in conflict zones where explosive devices and shrapnel, often heavily contaminated, pose a significant risk of life-threatening infections. Immediate and proper debridement, ideally conducted in an operating theatre in the presence of senior orthopaedic and plastic surgeons, is crucial for preventing severe complications and planning for subsequent reconstruction [35]. However, this review highlights that aligning with optimal care standards proved challenging, as specialist orthoplastic surgeons were often unavailable to oversee these critical procedures, attributed to the international volunteer shortfall and safety concerns [11,13,25]. By building field hospitals near the Turkish border, teams were able to establish communication to specialists in vascular, orthoplastics and cardiothoracics [23]. The composition of surgical teams, frequently made up of international volunteers or personnel from various organisations, underscores the necessity for a more structured and sustained global support mechanisms [23,25]. This would ensure that immediate and effective wound care becomes a more attainable standard in conflict-affected regions like Syria.

External fixation is widely regarded as the optimal method for stabilizing both open and closed fractures in conflict settings, given its adaptability to the unpredictable nature of battlefield injuries [36–42]. This surgical treatment for fractures involves bone stabilization via an external apparatus attached to bones via wires and rods that exit the skin. This technique is particularly effective for managing blast and GSW injuries, which often result in open wounds and multiple bone fragments [38]. The simplicity and cost-effectiveness of external fixators, requiring minimal parts and training, make them well-suited for conflict zones and accounted for 25.9% of surgical procedures here. Among the several models that exist, the Arbeitsgemeinschaft für Osteosynthesefragen (AO) modular was notably prevalent during the Syrian conflict, utilized in 585 (61.3%) cases. Given the importance of external fixators, provision of this equipment should be considered by governments and organisations when providing aid to conflict affected regions.

Hasanin et al. highlighted several of the operational challenges impacting surgical outcomes, including the loss of electricity in operating facilities, which led to the spoilage of blood units and necessitated manual ventilation methods [23]. Additionally, shortages in essential surgical equipment, such as Fogarty catheters and suture materials, further complicated patient care. These logistical hurdles often required the transfer of complex cases to Turkish hospitals post-resuscitation, a process facilitated by a field hospitals’ proximity to the Turkish border [23,25]. The expedited setup of these field hospitals, while necessary, resulted in inadequate documentation practices. This not only hampered effective patient follow-up but also poses significant challenges for conducting retrospective analyses [28].

In considering the findings of this review, it is essential to acknowledge several limitations that may influence the interpretation of the data. The retrospective nature of studies introduces the potential for recall bias. Similarly, reliance on data from a conflict-affected country, where documentation can be sporadic and patient transfers between healthcare facilities are common, exacerbates the risk of incomplete data capture. Selection bias, particularly in the form of survivor bias, could skew results. Moreover, the destruction or abandonment of numerous health facilities within the conflict zone suggests that the surgical capabilities depicted in this review may well represent an underestimation of the actual conditions and challenges faced. The lack of more recent data from 2020 may have implications for interpreting the most up to date surgical needs.

It is important to recognise that using data from an active war zone raises complex ethical considerations. On one hand, such data can be invaluable for humanitarian efforts, providing up to date insights into the nature and volume of injuries allowing for prioritization and targeted care delivery. Delaying data collection until the conflict subsides would significantly hinder the timeliness and effectiveness of any response [43]. However, researchers must ensure that their work does not inadvertently legitimise or provide a platform for warring factions. Care must also be taken to avoid placing already vulnerable communities at additional risk, whether through the publication of sensitive findings or the methods used to collect data.

These findings underpin the urgent need for increased investment in surgical care and robust international support and research in conflict zones. The pre-conflict state of Syria’s healthcare system, already strained, has been severely compromised, requiring comprehensive international aid. The significant toll on civilians, emphasizes the need for accessible, high-quality reconstructive surgical care. Future strategies should focus on creating sustainable healthcare infrastructure, training programs and provision of basic medical equipment. Despite a large number of procedures performed, publications reporting on more recent surgical activity and on outcome data throughout the conflict was lacking. Developing harmonised health information systems for surgical procedures and related data registries are required to support future studies. These efforts could contribute to closing existing care gaps and enhancing global medical response to conflict-related healthcare challenges.

## Supporting information

S1 File Search strategies. (DOCX)

S1 Fig Results of quality assessment. (DOCX)

S1 Table Inclusion and exclusion criteria. (DOCX)

S1 PRISMA Checklist. Preferred Reporting Items for Systematic reviews and Meta-Analyses Extension for Scoping Reviews (PRISMA-ScR) Checklist .
(DOCX)
