# Supplementary material for: Peripheral extremity surgery performed during the Syrian conflict – A scoping review
Source: PLOS Glob Public Health. 2025 Feb 10;5(2):e0004116. doi: 10.1371/journal.pgph.0004116 (PMC11809873; doi:10.1371/journal.pgph.0004116)
Supplement: S1 File — (DOCX) [file pgph.0004116.s001.docx]

## **S1 File**. Search strategies

Detailed search strategy for MEDLINE, EMBASE, Global Health, SCOPUS, Web of Science, Cochrane library

### MEDLINE, via OVID

| **Term groups** | **#** | **Terms** | **Hits** |
| --- | --- | --- | --- |
| **Syria** | 1 | Syria/ or syria*.mp. | 15863 |
|  | 2 | levant*.mp. | 1537 |
|  | 3 | damas*.mp. | 1711 |
|  | 4 | aleppo.mp. | 335 |
|  | 5 | hal#b.mp. | 12 |
|  | 6 | latakia.mp. | 26 |
|  | 7 | homs.mp. | 175 |
|  | 8 | hama?.mp. | 2575 |
|  | 9 | raq?a*.mp. | 45 |
|  | **10** | **1 or 2 or 3 or 4 or 5 or 6 or 7 or 8 or 9** | **21647** |
| **Surgery type** | 11 | trauma.mp. or exp "Wounds and Injuries"/ | 1205210 |
|  | 12 | exp orthopedics/ or exp traumatology/ | 27642 |
|  | 13 | orthop?edic?.mp. | 152823 |
|  | 14 | musculoskeletal.mp. or exp Musculoskeletal System/ or exp Musculoskeletal Diseases/ | 2441590 |
|  | 15 | Vascular Surgical Procedures/ or vascular.mp. | 901880 |
|  | 16 | plastic.mp. or Plastics/ | 201643 |
|  | **17** | **11 or 12 or 13 or 14 or 15 or 16** | **4283035** |
| **Outcome** | **18** | **(surger* or procedure? or operation? or intervention? or perform* or treatment? or outcome? or effect?).mp.** | **18773030** |
| **Combined** | **19** | **10 and 17 and 18** | **1335** |
|  | **20** | **limit 19 to yr="2011-Current"** | **709** |

### EMBASE, via OVID

| **Term groups** | **#** | **Terms** | **Hits** |
| --- | --- | --- | --- |
| **Syria** | 1 | exp Syrian Arab Republic/ or syria*.mp. | 22296 |
|  | 2 | (levant* or damas* or Aleppo or hal#b or latakia or homs or hama? or raq?a).mp. | 9147 |
|  | **3** | **1 or 2** | **30775** |
| **Surgery type** | 4 | trauma.mp. or exp injury/ | 2842408 |
|  | 5 | orthop?edic?.mp. or exp orthopedics/ | 208163 |
|  | 6 | exp musculoskeletal injury/ or exp musculoskeletal system/ or musculoskeletal.mp. or exp musculoskeletal disease/ | 4056551 |
|  | 7 | vascular.mp. | 1261929 |
|  | 8 | plastic/ or plastic surgery/ | 95581 |
|  | **9** | **4 or 5 or 6 or 7 or 8** | **7016505** |
| **Outcome** | **10** | **(surger* or procedure? or operation? or intervention? or perform* or treatment? or outcome? or effect?).mp.** | **23006816** |
| **Combined** | **11** | **3 and 9 and 10** | **2821** |
|  | **12** | **limit 11 to yr="2011-Current"** | **1856** |

### Global Health, via OVID

| **Term groups** | **#** | **Terms** | **Hits** |
| --- | --- | --- | --- |
| **Syria** | 1 | syria*.mp. or exp Syria/ | 4104 |
|  | 2 | (levant* or damas* or Aleppo or hal#b or latakia or homs or hama? or raq?a).mp. | 2013 |
|  | **3** | **1 or 2** | **5827** |
| **Surgery type** | 4 | exp trauma/ or trauma.mp. | 52067 |
|  | 5 | orthop?edic?.mp. or exp orthopaedics/ | 6879 |
|  | 6 | exp musculoskeletal system/ or musculoskeletal.mp. | 107520 |
|  | 7 | vascular.mp. | 51881 |
|  | 8 | plastic.mp. | 13838 |
|  | **9** | **4 or 5 or 6 or 7 or 8** | **218218** |
| **Outcome** | **10** | **(surger* or procedure? or operation? or intervention? or perform* or treatment? or outcome? or effect?).mp.** | **2499643** |
| **Combined** | **11** | **3 and 9 and 10** | **183** |
|  | **12** | **limit 11 to yr="2011-Current"** | **152** |

### SCOPUS

| **Term groups** | **#** | **Terms** | **Hits** |
| --- | --- | --- | --- |
| **Syria** | 1 | TITLE-ABS-KEY ( syria*) AND PUBYEAR > 2010 | 14,213 |
|  | 2 | TITLE-ABS-KEY ( levant* OR damas* OR aleppo OR hal?b OR latakia OR homs OR hama? OR raq*a ) AND PUBYEAR > 2010 | 15,985 |
|  | **3** | **1 AND 2** | **39,301** |
| **Surgery type** | 4 | TITLE-ABS-KEY ( trauma) AND PUBYEAR > 2010 | 245,473 |
|  | 5 | TITLE-ABS-KEY ( orthop*edic*) AND PUBYEAR > 2010 | 125,362 |
|  | 6 | TITLE-ABS-KEY (plastic) AND PUBYEAR > 2010 | 481,369 |
|  | 7 | TITLE-ABS-KEY ( vascular ) AND PUBYEAR > 2010 | 580,707 |
|  | 8 | TITLE-ABS-KEY ( musculoskeletal ) AND PUBYEAR > 2010 | 133,442 |
|  | **9** | **4 AND 5 AND 6 AND 7 AND 8** | **1,566,353** |
| **Outcome** | **10** | **TITLE-ABS-KEY ( surger* OR procedure* OR operation* OR intervention* OR perform* OR treatment* OR outcome* OR effect* )** AND PUBYEAR > 2010 | **23,280,064** |
| **Combined** | **11** | **( TITLE-ABS-KEY ( surger* OR procedure* OR operation* OR intervention* OR perform* OR treatment* OR outcome* OR effect* ) ) AND ( ( TITLE-ABS-KEY ( trauma ) ) OR ( TITLE-ABS-KEY ( orthop*edic* ) ) OR ( TITLE-ABS-KEY ( musculoskeletal ) ) ) AND ( ( TITLE-ABS-KEY ( levant* OR damas* OR aleppo OR hal?b OR latakia OR homs OR hama? OR raq?a ) ) OR ( TITLE-ABS-KEY ( syria* ) ) )** AND PUBYEAR > 2010 | **519** |

### Web of Science

| **Term groups** | **#** | **Terms** | **Hits** |
| --- | --- | --- | --- |
| **Syria** | 1 | (TS=(syria*)) AND PY=(2011-2024) | 18,196 |
|  | 2 | (TS=(levant* or damas* or Aleppo or hal?b or latakia or homs or hama? or raq?a)) AND PY=(2011-2024) | 12,144 |
|  | **3** | **#2 OR #1** | **29,097** |
| **Surgery type** | 4 | (TS=(trauma)) AND PY=(2011-2024) | 207,208 |
|  | 5 | (TS=(orthop*edic*)) AND PY=(2011-2024) | 76,253 |
|  | 6 | (TS=(musculoskeletal)) AND PY=(2011-2024) | 64,809 |
|  | 7 | (TS=(plastic)) AND PY=(2011-2024) | 282,326 |
|  | 8 | (TS=(vascular)) AND PY=(2011-2024) | 435,137 |
|  | **9** | **#6 OR #5 OR #4** | **1,065,733** |
| **Outcome** | **10** | **(((((((TS=(surger*)) OR TS=(procedure*)) OR TS=(operation*)) OR TS=(intervention*)) OR TS=(perform*)) OR TS=(treatment*)) OR TS=(outcome*)) OR TS=(effect*)** | **18,256,428** |
| **Combined** | **11** | **#10 AND #9 AND #3** | **506** |

### Cochrane library

| **Term groups** | **#** | **Terms** | **Hits** |
| --- | --- | --- | --- |
| **Syria** | 1 | MeSH descriptor: [Syria] explode all trees | 63 |
|  | 2 | (syria*):ti,ab,kw | 271 |
|  | 3 | (levant* or damas* or Aleppo or hal?b or latakia or homs or hama? or raq?a):ti,ab,kw | 2432 |
|  | **4** | **#1 or #2 or #3** | **2653** |
| **Surgery type** | 5 | MeSH descriptor: [Wounds and Injuries] explode all trees | 35269 |
|  | 6 | (trauma):ti,ab,kw | 19378 |
|  | 7 | MeSH descriptor: [Orthopedic Procedures] explode all trees | 17742 |
|  | 8 | (orthop?edic*):ti,ab,kw | 15018 |
|  | 9 | MeSH descriptor: [Musculoskeletal Diseases] explode all trees | 57880 |
|  | 10 | (musculoskeletal):ti,ab,kw | 17426 |
|  | **11** | **#5 or #6 or #7 or #8 or #9 or #10** | **130990** |
| **Outcome** | 12 | (surger* or procedure? or operation? or intervention? or perform* or treatment? or outcome? or effect?):ti,ab,kw | 1745625 |
| **Combined** | **13** | **#4 and #11 and #12** | **141** |
|  |  | **With date restrictions Jan 2011 to Nov 2023** | **128** |
