# Supplementary material for: Peripheral extremity surgery performed during the Syrian conflict – A scoping review
Source: PLOS Glob Public Health. 2025 Feb 10;5(2):e0004116. doi: 10.1371/journal.pgph.0004116 (PMC11809873; doi:10.1371/journal.pgph.0004116)
Supplement: S1 Fig — (DOCX) [file pgph.0004116.s002.docx]

## **S1 Fig**. Results of Quality Assessment


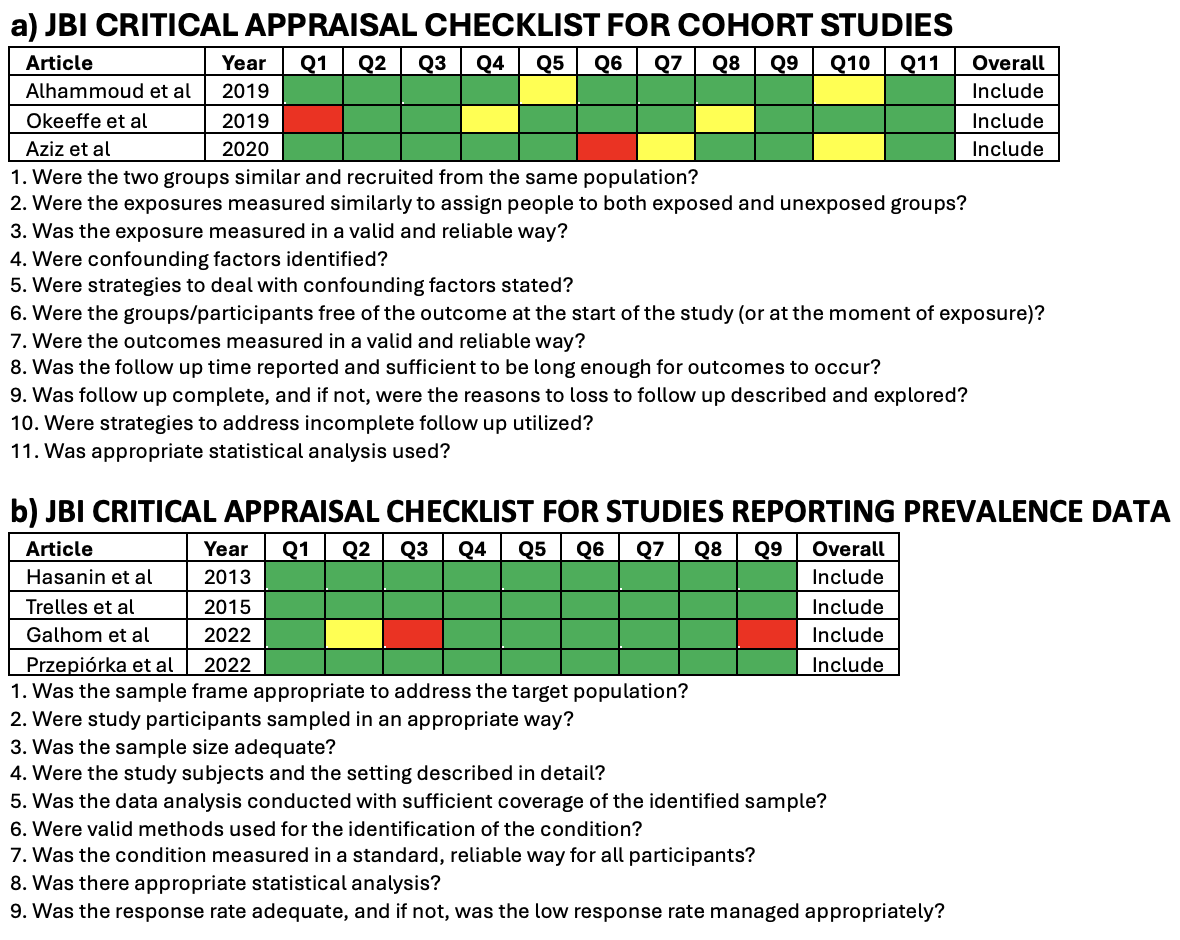


Results of the quality assessment using the Joanna Briggs Institute (JBI) critical appraisal tools for a) cohort studies b) prevalence data. Green = yes, yellow = unclear, red = no
