# Supplementary material for: Peripheral extremity surgery performed during the Syrian conflict – A scoping review
Source: PLOS Glob Public Health. 2025 Feb 10;5(2):e0004116. doi: 10.1371/journal.pgph.0004116 (PMC11809873; doi:10.1371/journal.pgph.0004116)
Supplement: S1 Table — (DOCX) [file pgph.0004116.s003.docx]

## **S1 Table.** Inclusion and exclusion criteria

Full data extraction table for the scoping review following selection of studies.

| **Subject** | **Included** | **Excluded** |
| --- | --- | --- |
| *Populations* | Syrian populations in Syria | Studies of populations outside of Syria. |
| *Interventions* | Include orthoplastics, vascular and peripheral limb procedures  Whole procedure must be performed within Syrian hospitals | Chemical warfare  Abdominal, ocular, cardiothoracic, neurosurgical, urological trauma |
| *Outcomes* | Any outcome on surgical procedures | Description of injuries/diseases only. |
| *Study designs* | Primary research of any quantitative or qualitative study design from published and grey literature | Non-scientific reports, letters and correspondences.  Reviews  Abstracts  Poster proceedings  Case reports |
| *Language* | English | Any other language |
| *Publication dates* | Between March 2011 (onset of the Syrian uprisings) and January 2024 | Any other dates |
